# Humidity‐Responsive RGB‐Pixels via Swelling of 3D Nanoimprinted Polyvinyl Alcohol

**DOI:** 10.1002/advs.202204469

**Published:** 2022-11-14

**Authors:** Byoungsu Ko, Jaekyung Kim, Younghwan Yang, Trevon Badloe, Jeonghoon Park, Joo Hwan Ko, Minsu Jeong, Hyunjung Kang, Chunghwan Jung, Young Min Song, Junsuk Rho

**Affiliations:** ^1^ Department of Mechanical Engineering Pohang University of Science and Technology (POSTECH) Pohang 37673 Republic of Korea; ^2^ School of Electrical Engineering and Computer Science Gwangju Institute of Science and Technology (GIST) Gwangju 61005 Republic of Korea; ^3^ Department of Chemical Engineering Pohang University of Science and Technology (POSTECH) Pohang 37673 Republic of Korea; ^4^ POSCO‐POSTECH‐RIST Convergence Research Center for Flat Optics and Metaphotonics Pohang 37673 Republic of Korea; ^5^ National Institute of Nanomaterials Technology (NINT) Pohang 37673 Republic of Korea

**Keywords:** Fabry–Pérot, nanoimprint, nanoparticle, polyvinyl alcohol, tunable coloration

## Abstract

Humidity‐responsive structural coloration is actively investigated to realize real‐time humidity sensors for applications in smart farming, food storage, and healthcare management. Here, humidity‐tunable nano pixels are investigated with a 700 nm resolution that demonstrates full standard RGB (sRGB) gamut coverage with a millisecond‐response time. The color pixels are designed as Fabry–Pérot (F–P) etalons which consist of an aluminum mirror substrate, humidity‐responsive polyvinyl alcohol (PVA) spacer, and a top layer of disordered silver nanoparticles (NPs). The measured volume change of the PVA reaches up to 62.5% when the relative humidity (RH) is manipulated from 20 to 90%. The disordered silver NP layer permits the penetration of water molecules into the PVA layer, enhancing the speed of absorption and swelling down to the millisecond level. Based on the real‐time response of the hydrogel‐based F–P etalons with a high‐throughput 3D nanoimprint technique, a high‐resolution multicolored color print that can have potential applications in display technologies and optical encryption, is demonstrated.

## Graphical abstract

Reversible humidity‐responsive 700 nm^2^ full‐color pixels are demonstrated through nanoimprinting lithography. The pixel consists of a mirror, humidity‐responsive polyvinyl alcohol, and top silver nanoparticles, and demonstrates the sRGB with the relative humidity changes. Silver nanoparticles shorten the modulation speed by a millisecond due to porosity, while the effective optical properties extend the absorption bandwidth, thereby enabling vivid color.

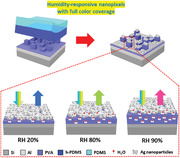

## 1 Introduction

Structural color metasurfaces have been actively investigated with the advantages of providing high‐resolution, a broader range of colors, lower toxicity, and long‐term durability compared to commercial pigment‐based coloration methods.^[^ ^1^, ^2^, ^3^ ^]^ To generate vivid colors, plenty of scattering mechanisms have been exploited with surface plasmon^[^ ^4^, ^5^, ^6^ ^]^ and Mie‐resonances.^[^ ^7^ ^]^ In addition, various materials including gold,^[^ ^8^ ^]^ silver,^[^ ^9^ ^]^ aluminum,^[^ ^10^ ^]^ hydrogenated amorphous silicon,^[^ ^11^, ^12^ ^]^ silicon nitride,^[^ ^13^ ^]^ gallium nitride,^[^ ^14^ ^]^ and titanium dioxide^[^ ^15^ ^]^ have been investigated. Recently, it has been shown that disordered plasmonic nanoparticles (NPs) alleviate the requirement of the top layer metal selectivity in Fabry‐Pérot (F–P) structures, that is to say, the optical properties are defined by the disorder of the NPs, rather than the material they are made of.^[^ ^5^, ^6^ ^]^ However, the previous coloration with fixed resonance frequencies has still limitations to their usage, as full‐color display devices require red, green, and blue (RGB) color pixels.

Tunable color metasurfaces have been steadily researched by exploiting functional materials that show changes in their optical properties when excited by an external stimulus. Various phase‐change materials such as Ge_2_Sb_2_Te_5_,^[^ ^16^, ^17^, ^18^ ^]^ Sb_2_S_3_,^[^ ^19^ ^]^ and VO_2_^[^ ^20^, ^21^, ^22^, ^23^, ^24^ ^]^ have been exploited. However, they cannot be used to create various colors in a single pixel because their optical properties are generally switched between only two distinct phases based on electron and lattice transition mechanisms.^[^ ^23^, ^25^ ^]^ On the other hand, liquid crystals (LCs) have been introduced to provide tunability through the polarization direction of the incident light on structural color metasurfaces composed of anisotropic nanostructures.^[^ ^26^ ^]^ In addition, pixel size of 2.1 µm has been demonstrated with polarization tuning with LCs.^[^ ^27^ ^]^ However, metasurfaces with LCs only exploit certain polarization states of the incident light so their brightness suffers as a result, and LC‐integrated color metasurfaces cannot be miniaturized to pixel sizes below one micron.^[^ ^28^ ^]^ Another mechanism of tunable color metasurfaces is using geometrically tunable nanostructures by changing its position, geometry, or volume.

Microelectromechanical systems (MEMS) have been applied to electrically tunable color metasurfaces,^[^ ^29^ ^]^ with diverse colors being demonstrated by changing the cavity size of F–P structures. Physical stretching mechanisms have also been exploited to change periodicity of the structures, leading to a large gamut coverage. However, MEMS‐integrated color metasurfaces cannot be easily employed to commercial display systems because the pixel size changes depending on the desired color. Another approach is grayscale lithography with volumetric tunable materials to create stepwise microstructures. Using this technique, various active color pixels have been demonstrated.^[^ ^30^, ^31^, ^32^, ^33^ ^]^ Here, we demonstrate a large‐scale reversible humidity‐responsive structural color platform through the single‐step nanoimprinting lithography (NIL) technique for 700 nm^2^ PVA pixels. The F–P pixels consist of disordered Ag NPs – humidity‐responsive PVA – Al mirror, and cover the full color gamut through change in the relative humidity (RH). Moreover, employing disordered Ag NPs enhances the modulation speed of the F–P structure due to the innate porous characteristics of NPs‐based films, while the effective optical properties of the Ag NPs extend the absorption bandwidth, thereby enabling the realization of vivid colors.^[^ ^34^ ^]^ To verify the proposed structure, we investigate the physical properties of the PVA films and Ag NPs. Then, we confirm the optical response of the F–P structure through numerical calculations and experiments. Finally, we demonstrate full‐color imaging with an image of a humidity‐responsive chameleon. The proposed device proves a profound improvement in terms of productivity due to both the single‐step NIL and lack of top metal deposition processes. We expect that this platform can be extensively applied to photonic devices such as colorimetric humidity sensors, high‐resolution displays, and optical security.

## 2 Results and Discussion

### 2.1 Rapid Humidity‐Responsive F–P Etalon

**Figure 1:**
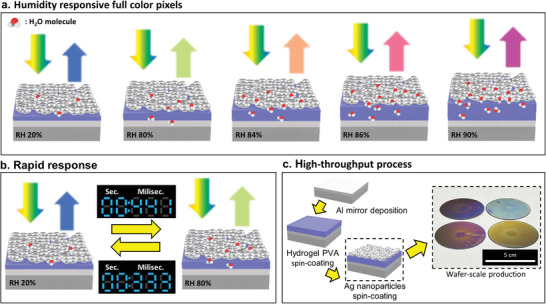
Schematics of our PVA Fabry–Pérot (F–P) etalon and its usages for full‐color pixels. a) Schematic of the humidity‐responsive F–P etalon, which changes reflective spectrum depending on the RH. b) Millisecond response time of the F–P etalon when the relative humidity is changed from 20 to 80%. The swelling and deswelling times are 441 and 333 ms, respectively. c) The F–P etalon is simply fabricated with one deposition and two spin‐coating processes, achieving wafer‐scale fabrication.

Recently, tunable coloration has been investigated using F–P etalons with various hydrogels (e.g., chitosan, PVA, etc.),^[^ ^34^, ^35^, ^36^, ^37^ ^]^ enabled by the physical modulation of the cavity size due to the swelling characteristics of the hydrogel (**Figure** **1**a). The generated color is modulated depending on the RH without the need for any extra external energy input. However, as they consist of metal–hydrogel–metal configurations, they suffer from extremely slow response times as the water molecules that can be absorbed into the hydrogel must first pass through surface defects in the top metal layer or from the side of the films. The previous research reveals the response time can be improved using porous Ag NPs–chitosan–Ag configuration.^[^ ^34^ ^]^ However, the chitosan film is formed by a two‐step process, which is composed of solution coating and deprotonation. This reason can make it challenging to apply for facile and high‐throughput fabrication. Therefore, we exploit the F–P etalon configuration which is composed of PVA and disordered Ag NPs for improving overall characteristics, such as the response time and fabrication process. To shorten the response time while maintaining a large color gamut, we employ disordered Ag NPs as the top layer, which significantly improves the penetration speed of the water molecules into the PVA layer. The measured swelling and deswelling time approaches 441 and 333 ms, respectively, with a gamut that is comparable to sRGB (Figure 1b) (Detailed comparisons are provided in **Table** **1**). In addition, our designed structure delivers additional fabrication benefits as it can be simply fabricated through two spin‐coating processes. Wafer‐scale humidity‐responsive F–P etalons are demonstrated on commercial 2‐inch Si wafers (Figure 1c) and could be easily expanded further to larger‐sized wafers. To generate sub‐micron full color pixels, we employ a 3D nanoimprinting technique after the spin‐coating of the PVA.

**Table 1:** Comparisons of pixel size and response/recovery time with the previously reported humidity‐tunable F–P etalons

[Ref.] (Year)	Polymer	Top layer material	Pixel size [µm]	Response time [ms]	Recovery time [ms]
Ours (2022)	PVA	Ag NPs	0.7	441	333
^[^ ^34^ ^]^ (2022)	Chitosan	Ag NPs + SCN ligands	2	119	107
^[^ ^34^ ^]^ (2022)	Chitosan	Ag NPs + OLA ligands	2	304	394
^[^ ^34^ ^]^ (2022)	Chitosan	Ag film	N/A	3,800,000	3,000,000
^[^ ^35^ ^]^ (2019)	Chitosan	Ag film	N/A	3,800,000	3,000,000
^[^ ^38^ ^]^ (2019)	Cellulose	Au film	N/A	600,000	600,000
^[^ ^32^ ^]^ (2021)	PVA	Pt film	5	—	—
^[^ ^31^ ^]^ (2022)	PVA	Ag film	2	≈10,000	≈10,000
^[^ ^39^ ^]^ (2021)	Poly‐acrylamidobenzophenone	Au film	N/A	21,600,000	21,600,000

### 2.2 Characterization of PVA Swelling and Ag NP Index

**Figure 2:**
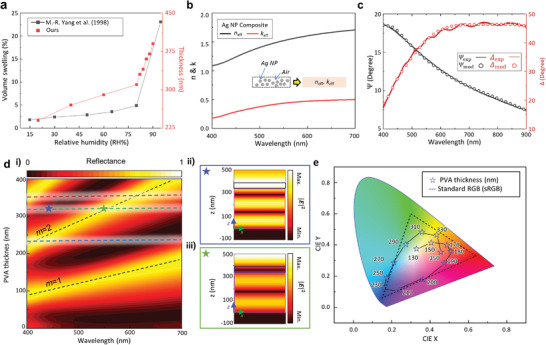
Design of PVA Fabry–Pérot (F–P) etalon with Ag NPs and Al mirror. a) Estimated volume swelling of spin‐coated PVA films (black dots) and its comparison with the previously reported (red dots).^[^ ^40^ ^]^ b) Measured effective refractive index *n*_eff_ and extinction coefficient *k*_eff_ using commercial ellipsometry. The Ag NP films are assumed to be a composite of spherical Ag NPs in air. c) The measured and modeled Fresnel parameters of the ellipsometry data. The model (dots) coincides closely with the measured data (lines), confirming the reliability of measured optical properties of Ag NPs. *Ψ*_exp_: Measured amplitude ratio; *Ψ*_mod_: Modeled amplitude ratio; *Δ*_exp_: Measured phase difference; *Δ*_mod_: Modeled phase difference. d‐i) Simulated reflectance spectra depending on the PVA thickness. Black dotted lines: guide of the order of resonance mode (*m*). Red, green, blue lines: thickness that relates to RGB colors. d‐ii,iii) Simulated electric field distribution at dip (ii) and peak (iii) of the green dotted line spectrum when *d* = 310 nm. e) Simulated colors plotted on the CIE 1931 chromaticity diagram for the various values of PVA thickness. The dashed line indicates the sRGB color gamut.

The swelling ratio of PVA is experimentally evaluated by retrieving the reflectance spectra of the humidity‐responsive F–P etalon (**Figure** **2**a). The thickness of the spin‐coated PVA slightly increases from 240 to 310 nm as the RH changes from 20 to 80%, while it changes dramatically when the humidity is adjusted from 80 to 90%, increasing from 310 to 390 nm. The measured PVA swelling trends agree well with previously reported values.^[^ ^40^ ^]^ The nonlinear relation of the PVA thickness and high RH can be explained by the storage modulus of PVA. With an increase in RH, the relaxation process of the polymer is accelerated by drastically absorbing water molecules, increasing the swelling rate along with the disruption rate of the hydrogen bonds.^[^ ^41^ ^]^ The optical properties of the Ag NPs (Ditto Technology CO.) films are obtained through ellipsometry by assuming it consists of spherical Ag NPs and air (Figure 2b). The Cauchy model is used to evaluate the complex refractive index of the Ag NPs while the filling ratio of the Ag NPs is estimated using the Maxwell–Garnet formula^[^ ^42^ ^]^ (Note S1, Supporting Information). Fresnel coefficients are measured by analyzing the polarization states of reflected light from a thin film using ellipsometry, and then the measured data is fitted. The filling ratio of the Ag NPs is estimated to be 8.5 wt%, which means that most of the Ag NP film consists of just air, which allows it to function as a porous film (Note S2, Supporting Information). The mean squared error between the measured and fitted data is 3.639, which confirms the reliability of the measured Ag NP film refractive indices (Figure 2c).

To confirm that the Ag NPs are not oxidized during deposition, X‐ray diffraction (XRD) pattern analysis is conducted. The measured XRD patterns include major diffraction peaks to 2*θ* = 38.10°, 44.28°, 64.41°, and 77.35°, which correspond to the [111], [200], [220], and [311] planes of Ag, respectively (Note S3, Supporting Information). As dispersion promoters (polymer) are coated on the Ag NPs, it is protected from being oxidized, enabling a long lifetime of the Ag NPs based devices.

The PVA exhibits a near‐zero extinction coefficient and its refractive index approaches 1.5 over whole visible frequencies (Note S4, Supporting Information). Although the refractive index should change depending on the amount of absorbed water molecules, we assume the PVA to have a constant refractive index at a different RH between the 20 and 80% as since both water and PVA exhibit a similar refractive index at the visible frequencies.

### 2.3 Full‐Color Gamut With the Humid‐Responsive F–P Etalon

We design and fabricate the F–P absorber structure through the use of thin‐film interference in the PVA cavity layer with the Ag NPs as the semi‐transparent mirror for absorption and reflection. First, to determine the initial color, we perform numerical calculations using rigorous coupled‐wave analysis (RCWA) for PVA thicknesses from 0 to 400 nm (Figure 2d‐i). The other variables, namely the Ag NPs layer and optically thick Al mirror are fixed at 70 and 150 nm, respectively. When the Ag NPs are coated at a high rpm (3,000 rpm), the surface roughness is limited.^[^ ^43^ ^]^ We achieve a 70 nm layer with a surface roughness of ≈30 nm, which is measured in ellipsometry (the effect of surface roughness will be discussed in Section 2.5). In addition, the 150 nm‐thick Al back reflector does not allow transmission of light, achieving a perfect reflective F–P interferometer in visible frequency.^[^ ^44^ ^]^ As PVA thickness increases, the resonance wavelength is red‐shifted owing to interference that can be expressed as:^[^ ^45^ ^]^

(1) $$d=\frac{\lambda_{\mathrm{res}}}{4n}$$

where *d* is the PVA thickness, *n* is the PVA refractive index (≈1.5), and *λ*_res_ is the resonance peak. For *d* of less than 50 nm, no noticeable interference is produced; and therefore, the reflectance over all visible wavelengths is similar. A reflection peak is formed by generating a 1st‐order resonance mode (*m* = 1) around *d* = 100 nm, which starts to enable the production of colors. As the *d* increases, higher‐order resonance modes (*m* = 2, 3, ⋅⋅⋅) are achieved, which produce vivid colors by creating a noticeable selective reflection peak. In the case of *d* = 320 nm, the reflectance dip is located at 450 nm and the peak is formed at 560 nm. This phenomenon can be observed from the electric field distribution simulation of the vertical cross‐section of the structure (Figure 2d‐ii,iii), which demonstrates a second‐order standing wave at the reflection peak and a non‐integer number of standing waves at the dip, producing destructive interference. The achievable color gamut for different *d* is comparable to the sRGB gamut (Figure 2e).

### 2.4 Tunable Responses Analysis of Humid‐Responsive F–P Etalon

**Figure 3:**
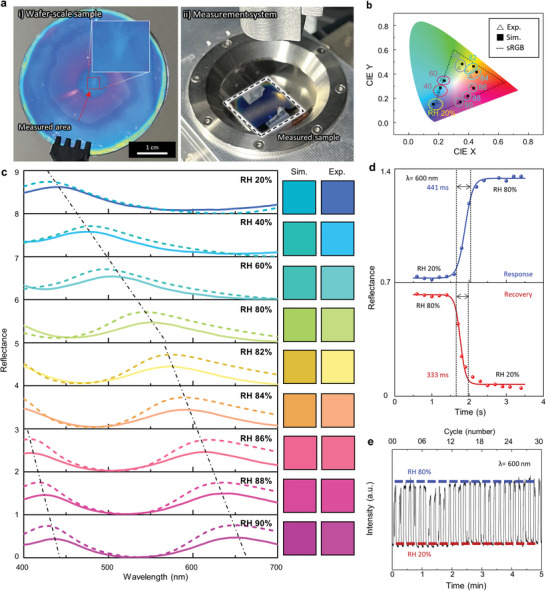
Demonstration of the PVA F–P etalon. a‐i) A wafer‐scale humid‐responsive F–P etalon is demonstrated and ii) its reflectance spectrum is measured. b) Simulated and experimentally measured color coordinates plotted on the CIE 1931 chromaticity diagram. c) Simulated and experimentally measured reflectance spectra from 400 to 700 nm by varying the RH from 20 to 90% with an initial PVA thickness of 219 nm. d) Response and recovery time plots and e) repeatability of the humidity‐responsive F–P etalon.

Based on the PVA spin‐curve, discussed in Note S5, Supporting Information, we deposit the corresponding thickness of PVA to produce wafer‐scale humid‐responsive F–P etalons (**Figure** **3**a‐i) that generate the desired colors. The calculated and measured reflectance spectra can be seen at each humidity, validating the accessible color range of the humid chamber and spectrometer (Figure 3a‐ii). The color over the whole wafer is not entirely consistent, which we attribute to miscalibration of the spin‐coater. This can be improved by size optimizing its chuck.

The reflected colors of the PVA F‐P etalon with different thicknesses are plotted on the CIE 1931 chromaticity diagram, revealing humidity‐responsive F‐P etalon (Figure 3b). Various samples with different initial PVA thicknesses are investigated according to the PVA concentration and spin‐curve (Notes S6 and S7, Supporting Information). An initial thickness of 219 nm is selected to obtain full‐color coverage by changing the RH from 20 to 90% (Figure 3c). Spectral analysis of the reflectance of the F–P etalon matches well with the simulated results. Minor discrepancies between numerical calculations and measurements can be attributed to differences in surface roughness of the NPs, slight mismatches in the thickness of the PVA layers, and swelling due to external environmental factors such as the RH at the time of measurement. To avoid water condensation on the chamber glass, we limit the maximum RH of the chamber as 90% (Details in Experimental Section). The measured reflectance peaks are red‐shifted as RH increases from 20 to 90%. The measured peak shifts around 1.8 nm per RH 1% but dramatically increases when the RH is modulated from 80 to 90%, to around 3.7 nm per RH 1%. The color difference between measured and sRGB is analyzed by using the color difference (*ΔE*_94_) concept. The *ΔE*_94_ is the weighted Euclidean distance in the *L*, *a*, *b* space with rectangular coordinates lightness, Chroma, and Hue.^[^ ^46^ ^]^ We have accomplished the full color by using a single F–P etalon depending on the varying humidity conditions. The red, green, and blue colors are demonstrated at RH 86, 80, and 20%, which calculated *ΔE*_94_ of each color are 18.178, 24.5865, and 19.0258, respectively (Note S8, Supporting Information). Each *ΔE*_94_ value gets closer to 0; color difference cannot be perceived by the human eye between the sRGB and measured color.

The response/recovery time can be explained as the difference in time required to reach from 10 to 90% (*T*_10–90_) and 90 to 10% (*T*_90–10_) intensity of each equilibrium state. The response and recovery time of the F–P etalon are measured by analyzing the reflectance spectra, achieving 441 ms response and 333 ms recovery times when the RH is varied between 20 and 80% (Figure 3d). These values are ≈20–30 times faster than that of previously reported PVA‐based devices with bulk metallic top layers.^[^ ^31^ ^]^ In contrast, our randomly distributed Ag NPs act as a porous film, which helps to accelerate the water molecular infiltration into the active PVA layer. This well‐known phenomenon is called Knudsen diffusion and occurs when the pore size is larger than the gas molecule and less than the mean free path of the gas molecule.^[^ ^47^ ^]^ Furthermore, the repeatability of our F–P etalon is also verified by repeating 30 cycles of the RH between 20 and 80% (Figure 3e). The peak intensity of the reflectance at 600 nm is consistently achieved over the 30 cycles, while quickly recovering back to the dip of low intensity at RH 20%.

### 2.5 3D Imprinted Humidity‐Responsive F–P Etalon for Full‐Color Displays

**Figure 4:**
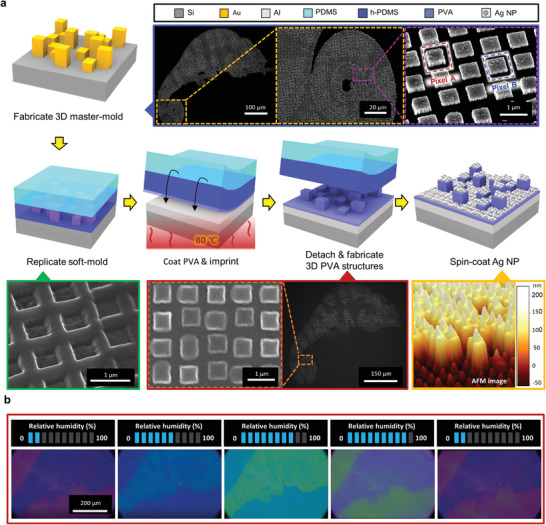
Full‐color pixels with 3D imprinted PVA F–P etalon. a) Fabrication process of 3D sub‐micron pixels. b) Real‐time reversible color rendering via external RH manipulation.

To demonstrate full‐color pixels, we use a single‐step nanoimprint lithography technique (**Figure** **4**a). To generate the multicolor pixels, a master mold, consisting of two cuboids with different heights, is fabricated using multilayered electron‐beam lithography (Note S9, Supporting Information). We attempted various colorations through the chameleon mold (600 µm^2^), which is made up of 250 nm (related to Pixel A) and 100 nm (related to Pixel B) height cuboids. Moreover, due to the nature of the NIL process, not only the transferred different thickness cuboid but also an extra residual layer is formed. In other words, each pixel and the residual layer can act as an individual resonator depending on the PVA thickness, hereby expressing three different colors (Note S10, Supporting Information). In addition, the PVA height can be controlled by O_2_ reactive ion etching,^[^ ^48^ ^]^ providing precise modulation of each pixel height to obtain the desired colors before coating with Ag NPs.

In addition, we employ 700 nm^2^ pixels for achieving a high resolution. However, for the sub‐micron pixels, it is challenging to measure by spectrometry, the reflectance of the pixel itself. Therefore, we considered the varying cuboid size effect of pixels which contained the residual layer (Note S11, Supporting Information). The calculation was conducted at cuboid diameters set with 100 to 900 nm for 280 nm height, which corresponds to Pixel B based on the 1 µm unit cell. In the unit cell, when the cuboid size gets smaller, the residual layer filling ratio will be larger, thereby the measured spectra could be distorted by the dominated area ratio. In addition, the cuboid size gets smaller, the pixel representing the nanostructure‐based optical response.^[^ ^49^ ^]^ These factors violate the height‐dependent F–P color mechanism and affect coloration. In addition, the overlay technique can be fabricated up to a sub‐20 nm distance from different nanostructures;^[^ ^50^ ^]^ however, when high humidity is exposed to PVA, structural interference based on over 20% volume swelling may occur.^[^ ^40^ ^]^ The cuboid pixel size was determined for considering these restrictions.

The single‐step color print process starts by replicating the stepwise thickness master mold as the soft mold. Low viscosity hard polydimethylsiloxane (h‐PDMS) is directly coated onto the master mold to fill the space, then cured.^[^ ^51^ ^]^ For smooth handling of the h‐PDMS layer, the soft mold is completed by pouring PDMS on the top of the h‐PDMS and curing it. The PVA resin is coated onto the hydrophobic‐processed soft mold and then transferred to an O_2_ plasma‐treated hydrophilic Al substrate under mechanical pressure. After the imprinting process, the PVA‐based stepwise nanostructures are released from the soft mold and are successfully transferred to the substrate due to the hydrophobic interaction (Details in Experimental Section).

Using this 3D nanoimprinting method, we experimentally demonstrate a humidity‐responsive F–P etalon with full color (Figure 4b). The imprinted cuboid heights of Pixel A and B are 430 and 280 nm, respectively, with a 200 nm thick residual layer. These different heights form the different reflective colors with a resonant peak shift. The resonance peak is not influenced much by the incident angles up to 30° (Note S12, Supporting Information).

The two different height structures (denoted as Pixel A and Pixel B in Figure 4a) are represented by cyan and blue colors, while the residual layer expresses magenta at the initial humidity condition (RH 20%). When the meta‐atoms are swollen, its swelling direction is mostly vertical (Note S13, Supporting Information). The measured spectra are confirmed by comparing with RCWA calculations as in the previous section (Note S6, Supporting Information). We note that the non‐uniformity of the PVA thickness can affect the F–P structural color. Discrepancies in the measured and calculated spectra can also originate from the surface roughness due to the top layer NPs, which form a non‐uniform film with disordered 20–40 nm Ag NPs. For a deeper analysis, we adopt the concept of root‐mean‐square (RMS) related to surface roughness. A value of 29.8 nm RMS is confirmed in the process of measuring the refractive index of the Ag NP film. According to the thin‐film interference theory, this thickness non‐uniformity is enough to display color mismatches in the localized pixels region.

For achieving full‐color, stepwise humid conditions (RH 20 to 90%) were applied to the humid‐responsive F–P etalon‐based micro‐image (Figure 4b). According to the previous section, it denotes that each pixel can achieve a full‐color rendering within RH 20 to 90%. During the changes in RH, the chameleon changes color and can be recovered back to its original state when the RH is modulated back to 20% (Video S1, Supporting Information). These tunable characteristics expand the potential of the humid‐responsive F–P etalon and are particularly feasible to be employed as sensors and smart labels.

## 3 Conclusion

In conclusion, we demonstrate a humidity‐responsive F–P etalon based on the inclusion of a layer of PVA between an Al mirror and Ag NP top layer. First of all, we introduced a single‐step NIL process for high‐throughput fabrication. With the ability to simply transfer high‐resolution patterns using NIL, we established a multi‐step F–P platform using PVA. The multi‐step pixels not only provide rich structural colors with sub‐micron resolution but also validate the high‐throughput fabrication technique, along with a rapid response to RH. Each pixel has a size of 700 nm^2^, and one pixel can generate red, green, and blue colors when the RH is modulated between 20 and 90%. Considering that F–P etalons have been steadily exploited for encryption devices working at visible frequencies, our humidity‐responsive F–P etalons are a promising candidate for designing functional displays within the field of metaphotonics.^[^ ^52^, ^53^, ^54^, ^55^ ^]^ We believe that our approach will help expand the degrees of freedom of conventional tunable F–P applications.

## 4 Experimental Section

### Optical Characterization

Optical properties of PVA and Ag NPs were obtained using the ellipsometer (M‐2000D, J. A. Woollam Co., USA). Humidity‐responsive optical characteristics (reflectance, repeatability, and reaction time) were measured in a custom‐made chamber equipped with a humidity sensor and UV–VIS–NIR spectrometer at a normal incidence. The RH was controlled by adjusting the flow rate defined by the water‐saturated nitrogen (N_2_) input and exhaust quantity in the chamber.

### Numerical Simulation

Numerically calculated results were obtained using the open‐source RCWA solver, “MAXIM”.^[^ ^56^ ^]^ The refractive index of Al was obtained from the Palik library, the measured refractive index of PVA and Ag NPs was used throughout. The sRGB color conversions were performed using the open‐source site “EasyRGB” accessed 20 April 2022.

### Overlay Master‐Mold Fabrication

The 3D master mold for the 3D humid‐responsive F–P nanoimprinting was fabricated using an overlay process with standard electron‐beam lithography (EBL), (Elionix, ELS‐7800, 80Kv, 100pA). First, Au alignment marker patterns were fabricated on a 500 µm‐thick silicon substrate with bilayer electron beam resist (PMMA 950 A2, and EL8). The first layer (EL8) was spin‐coated at 5,000 rpm for 60 s, and then the second layer (PMMA 950 A2) was spin‐coated at 2,000 rpm for 60 s. Each spin‐coated resin (EL8, and PMMA 950 A2) was baked at 150 °C for 5 min (EL8), and 180 °C for 5 min (PMMA 950 A2). Next, the substrate was cooled for 70 s, followed by spin‐coating of a conductive polymer layer (Showa Denko, E‐spacer 300Z) on top of the electron beam resist bilayer at 2,000 rpm for 60 s to prevent the charging effect from the dielectric layer. After the exposure process, the conductive layer was removed using de‐ionized (DI) water, and the electron beam resist was developed in MIBK:IPA 1:3 solution for 15 min. A 50 nm‐thick Au layer with a 3 nm Cr adhesion layer was deposited using an electron‐beam evaporator (KVT, KVE‐E4000), followed by a lift‐off process of dipping in acetone for 15 min at 75 °C and sonication process of 45 Hz for 4 min. Using the same EBL exposure, development, lift‐off conditions as the Au alignment marker, two different Au height structures were fabricated using the mark as an aligner. PMMA 495 A6 was used for the electron‐beam resin for easy Lift‐off process when the structures were made of 5 nm‐thick Cr, and 250 nm‐thick Au. Spin‐coating rate was 1,000 rpm for 60 s, resulting in a 600 nm‐thick layer. Following the first overlay process, using the same conditions of the first overlay process except for the deposition (5 nm‐thick Cr adhesion layer and 150 nm‐thick Au patterns) process and thickness of electron beam resist layer (Microchem, 495 polymethyl methacrylate [PMMA] A6 spin‐coated for 3,000 rpm for 60 s resulting in a 350 nm‐thick layer), the Au patterns of the second EBL overlay process were completed.

### Soft‐Mold Fabrication

Before replicating the nanostructures, the fabricated master mold was treated with an O_2_ plasma process to decontaminate it and improve hydrophilicity. The surface‐treated master mold formed a self‐assembled monolayer via a vaporized silane coupling agent (Trichloro‐1H, 1H, 2H, 2H‐Perfluorooctyl‐silane, Sigma–Aldrich) coating for 5 min at 130 °C, thereby exhibiting a hydrophobic surface, which allowed for facilitated demolding. After the surface treatment of the master mold, h‐PDMS was prepared by blending 1.7 g of vinylmethyl copolymer (VDT731, Gelest), 9 µL of platinum‐catalyst (SIP6831.2, Gelest), 0.05 g of the modulator (2,4,6,8 – tetramethyl‐ 2,4,6,8 – tetravinylcyclotetrasiloxane, Sigma–Aldrich), 1 g of toluene, and 0.5 g of siloxane‐based silane reducing agent (HMS‐301, Gelest). The h‐PDMS was spin coated on the master‐mold at 2,000 rpm for 60 s, followed by a baking process at 70 °C for 2 h to harden the h‐PDMS. Then, the PDMS mixture consisting of a 1:10 weight ratio of PDMS (Sylgard 184 A, Dow corning) and curing agent (Sylgard 184 B, Dow corning) was poured onto the h‐PDMS layer, followed by baking at 70 °C for 2 h to harden the h‐PDMS. After this process, the hardened soft mold was demolded from the master mold and used for replication.

### PVA Preparation

The specific wt% of PVA solution was prepared using commercial PVA (Dongyang Syn Co.) granular particles dissolved and stirred in deionized water at 90 °C.

### PVA Nanoscale Pixel Printing

4 wt% PVA was spin‐coated at 2,000 rpm for 60 s on the soft mold, then imprinted on the silicon substrate which had a deposited 150 nm Al for 10 min at 5 bar at a temperature of 80 °C. After 10 min, the soft‐mold was detached from the substrate, transferring the PVA nanostructures onto the substrate. After detaching the soft mold, Ag NPs were spin‐coated at 3,000 rpm for 60 s onto the PVA nanostructures to fabricate the tunable color nanoscale pixels.

## Conflict of Interest

The authors declare no conflict of interest.

## Supporting information

Supporting Information

Supplemental Video 1

## Data Availability

The data that support the findings of this study are available from the corresponding author upon reasonable request.
