# Supplementary material for: Humidity‐Responsive RGB‐Pixels via Swelling of 3D Nanoimprinted Polyvinyl Alcohol
Source: Adv Sci (Weinh). 2022 Nov 14;10(2):2204469. doi: 10.1002/advs.202204469 (PMC9839877; doi:10.1002/advs.202204469)
Supplement: Supplementary file 1 — Supporting Information [file ADVS-10-2204469-s001.pdf]

ADVANCED SCIENCE
Open Access

Supporting Information

for *Adv. Sci.*, DOI 10.1002/advs.202204469

Humidity-Responsive RGB-Pixels via Swelling of 3D Nanoimprinted Polyvinyl Alcohol

*Byoungsu Ko, Jaekyung Kim, Younghwan Yang, Trevon Badloe, Jeonghoon Park, Joo Hwan Ko, Minsu Jeong, Hyunjung Kang, Chunghwan Jung, Young Min Song and Junsuk Rho**

Supporting Information

Humidity-Responsive RGB-Pixels *via* Swelling of 3D Nanoimprinted Polyvinyl Alcohol

Byoungsu Ko[†]*, Jaekyung Kim*[†] *Younghwan Yang*[†]*, Trevon Badloe, Jeonghoon Park, Joo Hwan Ko, Minsu Jeong, Hyunjung Kang, Chunghwan Jung, Young Min Song, Junsuk Rho*[*]
[†]*These authors contributed equally to this work*

B. Ko, J. Kim, Y. Yang, T. Badloe, J. Park, M. Jeong, H. Kang, Prof. J. Rho.
Department of Mechanical Engineering
Pohang University of Science and Technology (POSTECH)
Pohang 37673, Republic of Korea
[*]E-mail: jsrho@postech.ac.kr

J. H. Ko, Prof. Y. M. Song.
School of Electrical Engineering and Computer Science
Gwangju Institute of Science and Technology (GIST)
Gwangju 61005, Republic of Korea

C. Jung, Prof. J. Rho.
Department of Chemical Engineering
Pohang University of Science and Technology (POSTECH)
Pohang 37673, Republic of Korea

Prof. J. Rho.
POSCO-POSTECH-RIST Convergence Research Center for Flat Optics and Metaphotonics
Pohang 37673, Republic of Korea

Prof. J. Rho.
National Institute for Nanomaterials Technology (NINT)
Pohang 37673, Republic of Korea

This PDF file includes

Supplementary Note 1. Effective index of spin-coated Ag nanoparticles
Supplementary Note 2. Transmittance of spin-coated Ag nanoparticles and evaporated Ag thin films
Supplementary Note 3. X-ray diffraction of spin-coated Ag nanoparticles
Supplementary Note 4. Refractive index of the PVA
Supplementary Note 5. Thickness analysis of spin-coated 3wt% to 5wt% PVA films
Supplementary Note 6. Analysis of Ag NPs-PVA cavity resonator reflectance depending on the initial PVA thickness
Supplementary Note 7. Repeatability of Ag NP-PVA cavity resonators depending on the initial thickness
Supplementary Note 8. Analysis of color difference between the sRGB and measured color
Supplementary Note 9. Schematic of overlay nanopixel mold fabrication
Supplementary Note 10. Reflectance spectra depending on the stepwise PVA height of nanopixels
Supplementary Note 11. Size effect analysis of nanopixels
Supplementary Note 12. Effect of incident angle on reflectance spectrum
Supplementary Note 13. Swelling simulation of PVA structures

Supplementary Note 1: Effective refractive index of spin-coated Ag nanoparticles

The complex refractive indices of the Ag nanoparticles (NPs)-based film is obtained using commercial ellipsometry. It is assume that the Ag NP films are consist of spherical Ag NPs and air, and its ratio is estimated by fitting volume ratio of inclusions β using the following Maxwell-Garnett formula[1]:

$$\varepsilon_{film} = \varepsilon_{air} + \beta(\varepsilon_i - \varepsilon_{air}) + \frac{3\varepsilon_{air}}{\varepsilon_i + 2\varepsilon_{air} - \beta(\varepsilon_i - \varepsilon_{air})}$$

where ε_{film}, ε_{air} and ε_i represent complex permittivity of Ag NPs-based film, the air and inclusion material (Ag NPs), respectively. During the fitting, ε_i is estimated with the Lorentz model whose initial coefficients is set as silver, and ε_{air} is fixed as 1 to describe the permittivity of the air. The fitted β is obtained as 0.085 that is the filling ratio of Ag NPs in the film.

Supplementary Note 2: Transmittance of spin-coated Ag nanoparticles and evaporated Ag thin films

Transmittance spectra of Ag NPs and Ag thin-films are measured using a home-made setup. We compare a deposited Ag thin film with various composite films of different concentrations of Ag NPs. At comparable thicknesses, the spin-coated Ag film with lower wt% has higher transmittance at comparable thickness since a porous medium makes lower imaginary part of permittivity that pure Ag films.

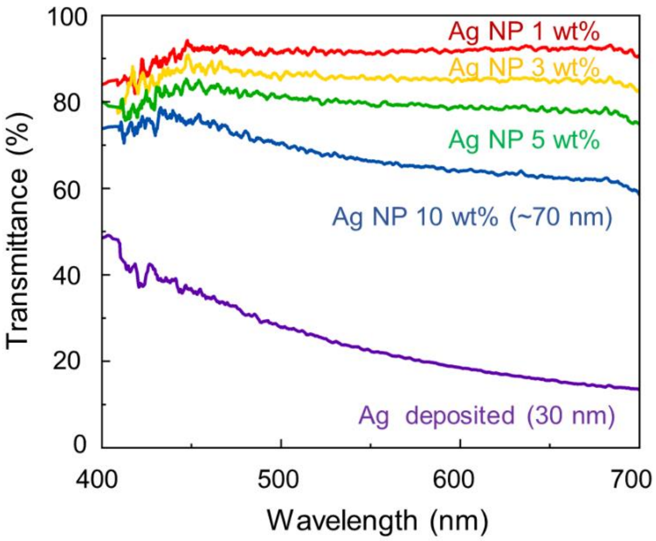

Figure S1. Measured Ag NPs and Ag film transmittance. The measured transmittance of evaporated Ag thin-films and spin-coated Ag NPs films fabricated under spin-coating at 3,000 rpm for 60 s using Ag NP concentrations of 1wt% (red), 3wt% (yellow), 5wt% (green), and 10wt% (blue).

Supplementary Note 3: X-ray diffraction of spin-coated Ag nanoparticles

X-ray diffraction (XRD) patterns are obtained to analyze Ag NPs composition and verify their non-oxidization during the fabrication processes. The sample was prepared by spin-coating 10 wt% Ag NPs at 3,000 rpm for 60 s on a glass substrate. Measured patterns confirms that Ag NPs does not have AgO and AgO_2.

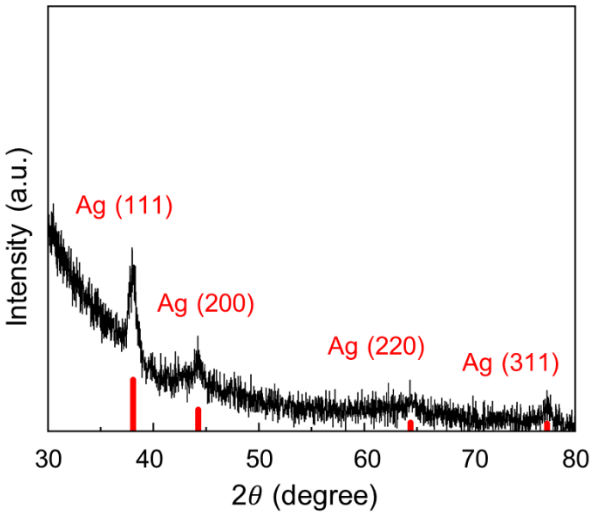

Figure S2. Analysis of X-ray diffraction (XRD) patterns of an Ag nanoparticles composite. The measured XRD pattern corresponds to the international center for diffraction data (ICDD) of Ag database.

Supplementary Note 4: Refractive index of the PVA

The PVA refractive index was measured with ellipsometry with a thin film, which 3wt% of PVA concentration coated at 3,000 rpm at 60 s. The complex refractive index has a similar tendency to a conventional polymer material that has an over the real value of 1.5, and a near-zero imaginary value (extinction coefficient).

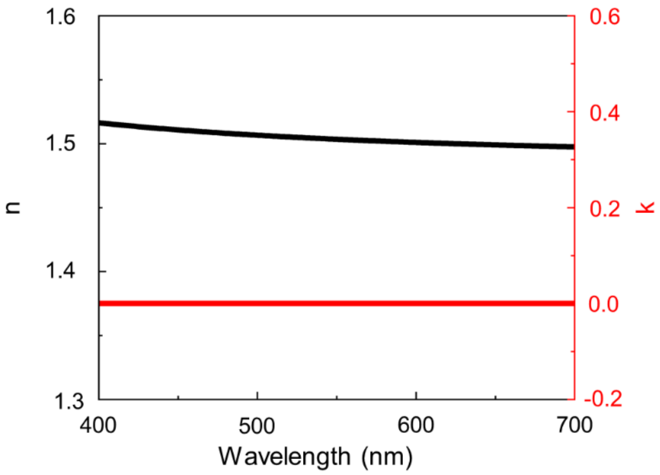

Figure S3. Refractive index of an PVA thin film. The complex refractive index consists of the real (black line) and imaginary value (red line). The measurement was conducted using ellipsometry (M-2000D, J.A. Woollam).

Supplementary Note 5: Thickness analysis of spin-coated 3wt% to 5wt% PVA films

The spin-coated PVA film thickness relies on the concentration at the same angular velocity. The volume of PVA molecules increases as the concentration of PVA increases, resulting in thicker films.

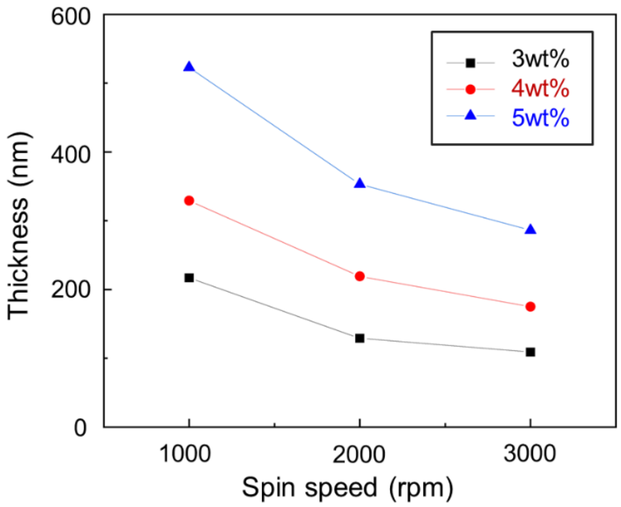

Figure S4. Measured spin-coated PVA film thickness. The measured thickness of PVA films spin-coated from 1,000 rpm to 3,000 rpm for PVA concentrations of 3wt% (black) 4wt% (red), and 5wt% (blue). The measurement was conducted using ellipsometry (M-2000D, J.A. Woollam).

Supplementary Note 6: Analysis of Ag NPs-PVA cavity resonator reflectance depending on the initial PVA thickness

The generated color is highly affected by the initial PVA cavity thickness, which is measured in atmospheric humidity conditions. Therefore, the direction of the color modulation in relation to the relative humidity (RH) is determined by the initial color. To attempt to analyze the color range, we fabricated various resonators by varying initial PVA thickness. Also, color coverage performance was confirmed by the reflectance spectra depending on the varying RH conditions.

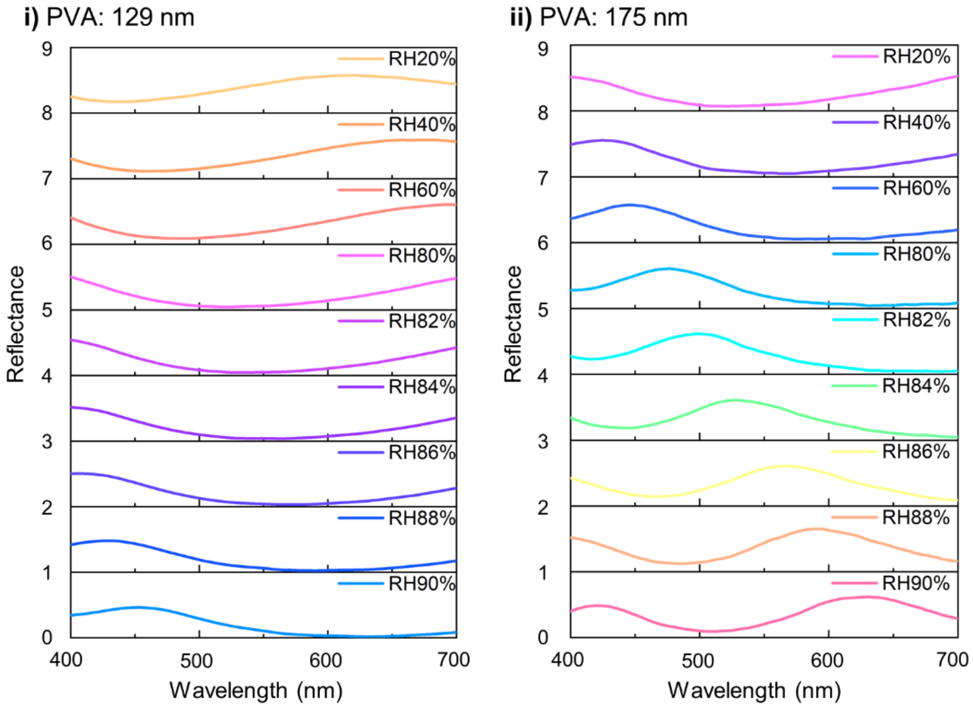

Figure S5. Measured reflectance of the humidity-responsive resonator. The reflectance spectrum was measured in i) 129 nm, and ii) 175 nm of initial PVA thickness conditions. Solid line color, measured color;

Supplementary Note 7: Repeatability of Ag NP-PVA cavity resonators depending on the initial thickness

Each structure has a different reflectance dip that is the result of the geometrical parameter and light interaction. The repeatability test is conducted at each reflection dip. The measurement consists of humidification and dehumidification by exposing the sample to H_2O saturated N_2 and dry air, respectively. Each step is performed for 5 s. We repeat 30 cycles between RH 20% and RH 80%, obtaining a repeatable approximate relative reflectance at each humidity state.

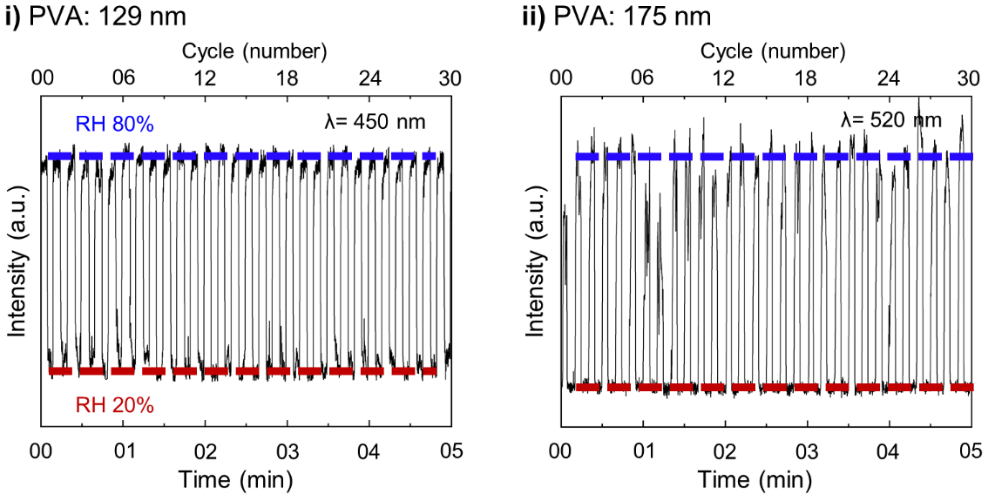

Figure S6. Measured repeatability of the humidity-responsive resonator. The measured repeatability cycle was measured at each reflection dip of i) 129 nm, and ii) 175 nm PVA thickness depending on the humidification (ON state: RH 80%) and dehumidification (OFF state: RH 20%) repeatedly.

Supplementary Note 8: Analysis of color difference between the sRGB and measured color

The color difference between the measured and sRGB was analyzed from ΔE_{94}, which improved from conventional ΔE. This concept employs the methodology of Euclidean distance of CIE coordinates which denotes L^*, a^*, b^* then can quantify the color difference. The color difference is based on the lightness (ΔL), chroma (ΔC_{ab}), and hue (ΔH_{ab}) calculated from L, a, b coordinates between the sRGB and measured color [2].

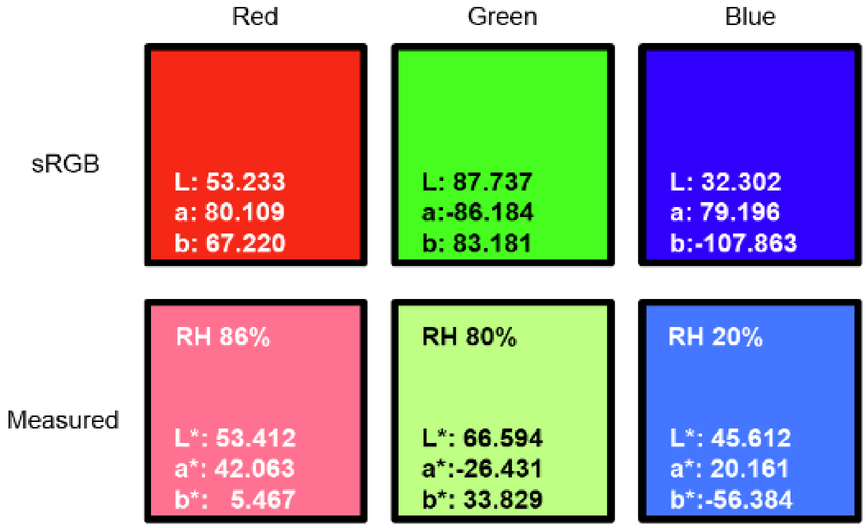

Figure S7. Reference sRGB and measured color coordinates.

The ΔE_{94} is express as:

$$\Delta E_{94} = \sqrt{\left(\frac{\Delta L}{K_L S_L}\right)^2 + \left(\frac{\Delta C_{ab}}{K_C S_C}\right)^2 + \left(\frac{\Delta H_{ab}}{K_H S_H}\right)^2} \cdots (1)$$

where each component of eq. (1) corresponds as follows:

$$\Delta E_{ab} = \sqrt{(L - L^*)^2 + (a - a^*)^2 + (b - b^*)^2}$$

$$\Delta C_{ab} = \sqrt{a^2 + b^2} - \sqrt{a^{*2} + b^{*2}}$$

$$\Delta H_{ab} = \sqrt{\Delta E_{ab}{}^2 - \Delta L^2 - \Delta C_{ab}{}^2}$$

$$S_L = 1,\ S_C = 1 + K_1(\sqrt{a^2 + b^2}),\ S_H = 1 + K_2(\sqrt{a^2 + b^2})$$

K_L=K_C=K_H=1 (for reference conditions)

From this calculation, each ΔE_{94} is quantified as 18.178, 24.5865, and 19.0258 at red, green, and blue respectively.

Supplementary Note 9: Schematic of overlay nanopixel mold fabrication

We employ an electron beam lithography (EBL) overlay technique to make two pixels with different thicknesses for the fabrication of the nanopixel master mold. The difference between typical EBL processes and an overlay process is that alignment marker patterns must be fabricated as reference points during the first and second overlay processes. For fabrication of the alignment marker patterns and nanopixels, an electron beam resist is spin-coated on the 500 μm-thick silicon substrate, followed by exposure and development process. Next, a Au layer and Cr adhesion layer are deposited using an electron beam evaporator, followed by a lift off process. After repeating these all processes twice, the fabrication of the nanopixel master mold is complete.

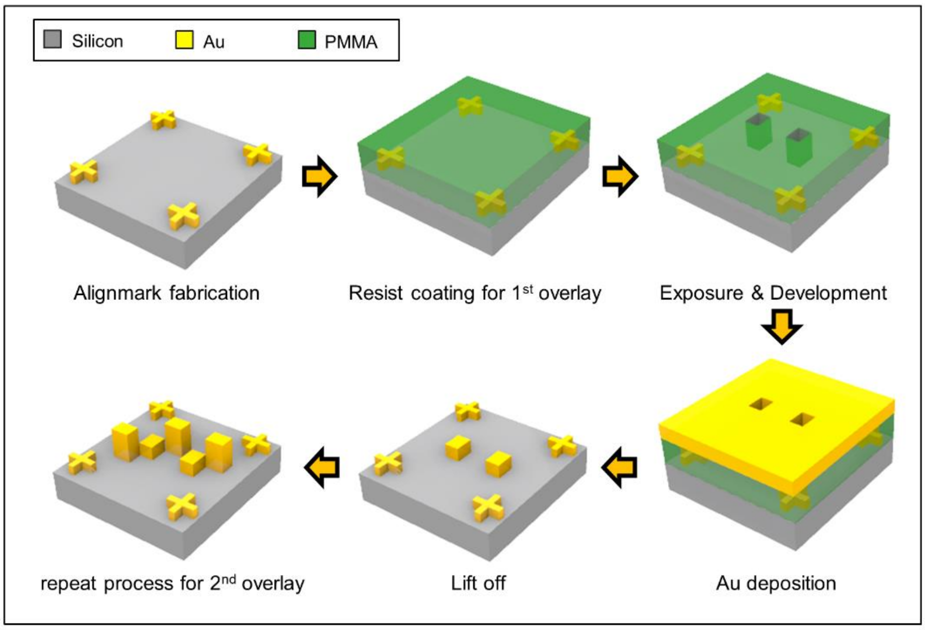

Figure S8. The overlay fabrication process for multilevel thickness master mold.

Supplementary Note 10: Reflectance spectra depending on the stepwise PVA height of nanopixels

The imprinted nanostructures are composed of different PVA heights, which are defined by the residue layer, Pixel A, and Pixel B. The imprinted residue thickness is about 200 nm, which contributes to an extra height for each pixel. Therefore, the PVA height of each Pixel A and B layer reached up to about 440 nm and 280 nm respectively. In the simulated spectra, the height of each Pixel A and B corresponded to 430, 260 nm. These discrepancies in the spectra can be explained by fabrication defects and surface roughness.

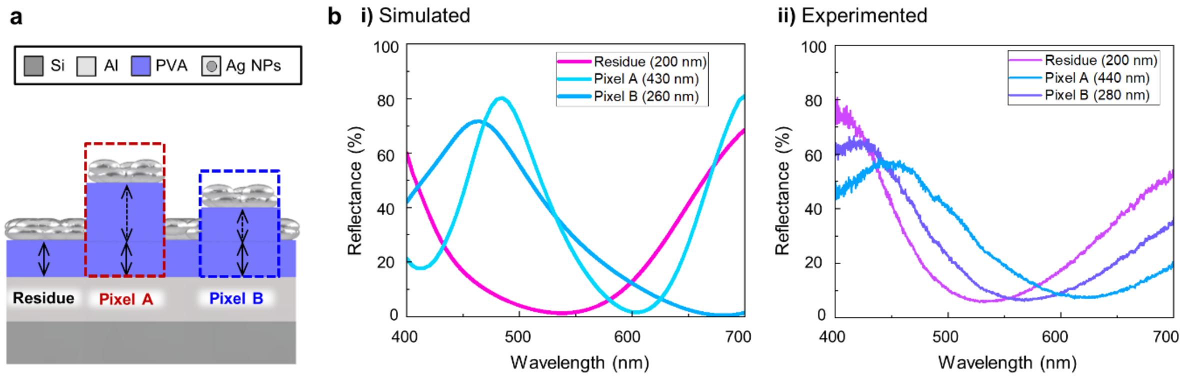

Figure S9. Simulated and experimented reflectance spectra of PVA nanopixels in initial humidity (RH 20%). (a) Cross-sectional image of humidity-responsive resonators. **(b)** Simulated and experimented reflectance spectra of each pixel.

Supplementary Note 11: Size effect analysis of nanopixels.

We investigated the varying cuboid size effect of pixels with contained the residue layer. The size effect simulation was conducted with cuboid size varying from 100 to 900 nm. The unit cell was fixed at 1 μm and the pixel's PVA height was fixed at 280 nm, which is corresponding to Pixel B height. The simulated spectra reveal that while the cuboid diameter gets approximated to 100 nm, the pixel's optical response will be reached to the nanodisc response, unwanted enhanced light-matter interaction such as extra reflection peak[3].

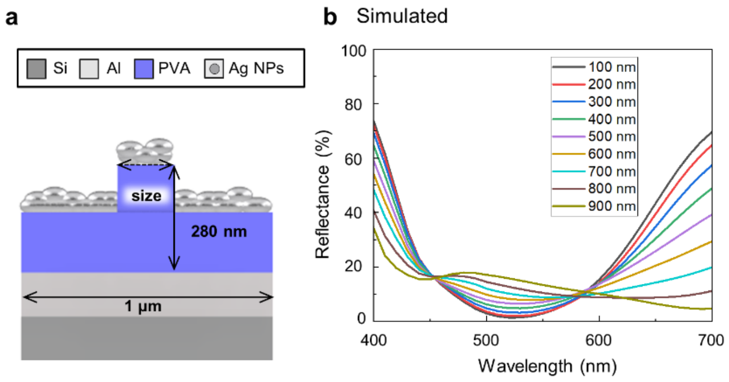

Figure S10. Simulated reflectance spectra of nanopixel. (a) Cross-sectional image of humidity-responsive resonators unit cell. **(b)** Simulated reflectance spectra of nanopixel depending on the size varying.

Supplementary Note 12: Effect of incident angle on reflectance spectrum

Reflectance spectra are calculated with rigorous coupled wave analysis when the incident angle is varied from 0° to 30° (**Figure S11**). Two cavity thicknesses (240, and 310 nm) are selected that represent the F-P etalon under RH 20% and RH 80%, respectively. Although peak intensities are decreased and blue-shifted as incident angle increases from 0° to 30°, the reflect color remains fairly consistent.

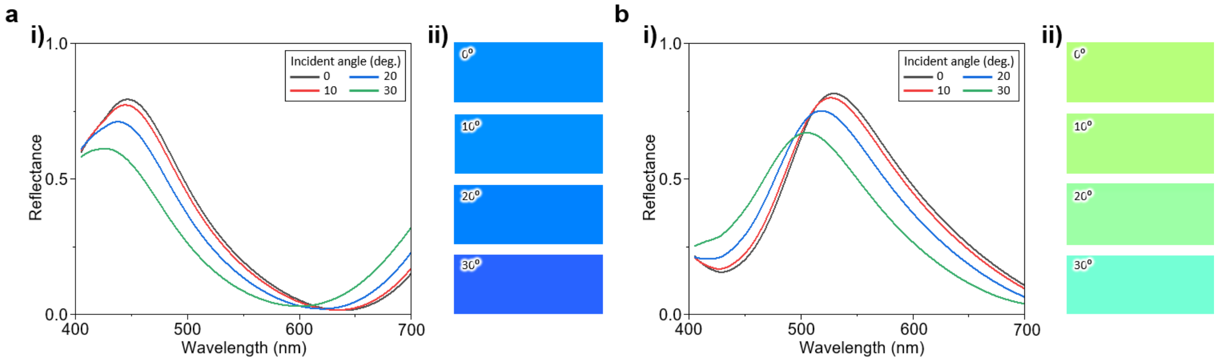

Figure S11. Reflectance at different incident angles. Reflectance spectra and color of the F-P etalon are calculated at a) RH 20% and b) RH 80%, for cavity thicknesses of 240 and 310 nm, respectively. i) The peak of the reflectance is slightly influenced by the incident angle, however ii) the reflected color remains similar to the designed color.

Supplementary Note 13: Swelling simulation of PVA structures.

PVA swelling simulations are conducted to verify the vertical expansion of PVA structures. We used hygroscopic swelling simulation with the equation of $\epsilon_{hy} = \beta_{hy}M_m(c\text{-}c_{ref})$, where ϵ_{hy} and β_{hy} are the hygroscopic strain and swelling coefficient, respectively; M_m is the molar mass of polymer; and c and c_{ref} are the atmospheric and reference concentrations of inclusion, respectively[4,5]. The substrate and Ag NPs are considered as fixed volume; PVA is swollen up to 35% and 30% for a film and meta-atoms, respectively. As a result, it is found that the vertical expansion is dominant direction in both PVA films and meta-atoms, resulting in cavity thickness variation with reflective color changes (**Figure S6**).

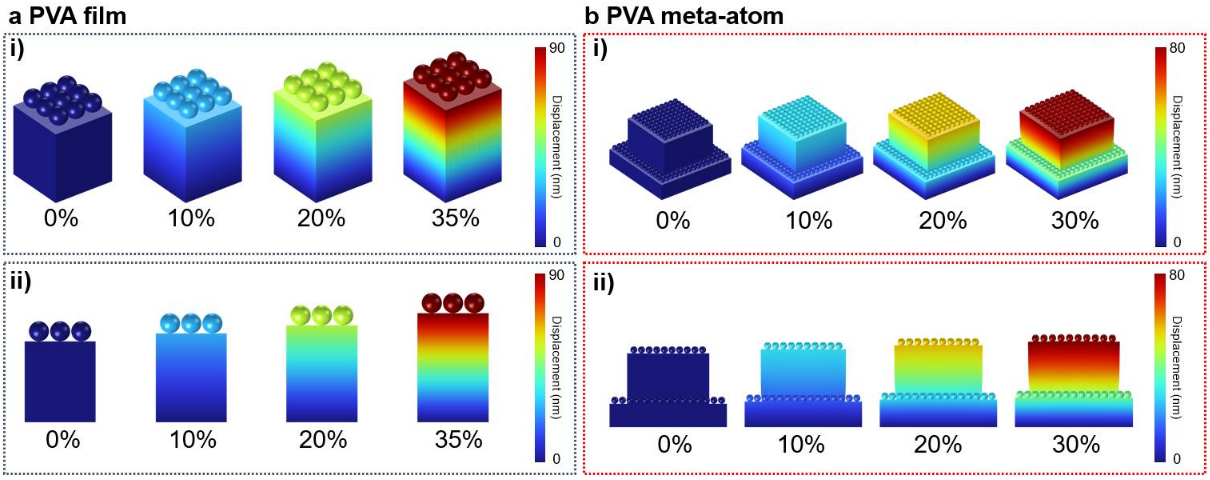

Figure S12. Swelling simulation of PVA structures. Swelling simulations are conducted on a) PVA films with the thickness of 240 nm, and b) meta-atom that have the same parameter with meta A. Schematic shows i) tilted view, and ii) side view. Rainbow color: displacement.

References

[1] V. A. Markel, *J. Opt. Soc. Am. A* **2016**, *33*, 1244.

[2] W. S. Mokrzycki, M. Tatol, *Mach. Graph. Vis.* **2011**, *Vol. 20, No. 4*, 383.

[3] R. Mudachathi, T. Tanaka, *Sci. Rep.* **2017**, *7*, 1199.

[4] Y. Yang, and Hong Zhao, *Appl. Surf. Sci.* **2022**, 557, 151895

[5] A. Ehrehofer, *Sens. Actuators B*, **2018**, 255, 1343-1353
